# Methyl 4-bromo-3-hy­droxy­benzoate

**DOI:** 10.1107/S1600536810051445

**Published:** 2010-12-11

**Authors:** Hua-Rong Huang, Zhi-Yun Du, Yu-Jing Lu, Yan-Xiong Fang, Kun Zhang

**Affiliations:** a Guangdong University of Technology, Faculty of Chemical Engineering and Light Industry, Guangzhou 510006, Guangdong, People’s Republic of China

## Abstract

In the title compound, C_8_H_7_BrO_3_, the meth­oxy­carbonyl group is twisted at a dihedral angle of 8.06 (4)° with respect to the benzene ring. In the crystal, mol­ecules are connected by O—H⋯O hydrogen bonds into helical chains running along the *b* axis.

## Related literature

For applications of methyl 3-hy­droxy­benzoate derivatives in the synthesis of various broad-spectrum anti­microbials, see: Zhong *et al.* (2001 ▶). For the synthesis of the title compound, see: Nie *et al.* (2005 ▶).
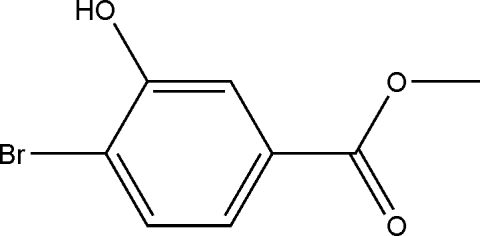


## Experimental

### 

#### Crystal data

C_8_H_7_BrO_3_
*M* *_r_* = 231.05
Monoclinic, 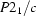

*a* = 10.812 (4) Å
*b* = 6.317 (2) Å
*c* = 12.490 (5) Å
β = 100.164 (6)°
*V* = 839.7 (5) Å^3^
*Z* = 4
Mo *K*α radiation
μ = 4.86 mm^−1^
*T* = 293 K
0.27 × 0.24 × 0.16 mm

#### Data collection

Bruker SMART CCD 1000 diffractometer
Absorption correction: multi-scan (*SADABS*; Sheldrick, 1996 ▶) *T*_min_ = 0.354, *T*_max_ = 0.511
4755 measured reflections
1831 independent reflections
1129 reflections with *I* > 2σ(*I*)
*R*_int_ = 0.042

#### Refinement

*R*[*F* ^2^ > 2σ(*F* ^2^)] = 0.058
*wR*(*F* ^2^) = 0.172
*S* = 1.08
1831 reflections
111 parameters
H-atom parameters constrained
Δρ_max_ = 0.46 e Å^−3^
Δρ_min_ = −0.55 e Å^−3^

### 

Data collection: *SMART* (Bruker, 1999 ▶); cell refinement: *SAINT-Plus* (Bruker, 1999 ▶); data reduction: *SAINT-Plus*; program(s) used to solve structure: *SHELXS97* (Sheldrick, 2008 ▶); program(s) used to refine structure: *SHELXL97* (Sheldrick, 2008 ▶); molecular graphics: *SHELXTL* (Sheldrick, 2008 ▶); software used to prepare material for publication: *SHELXTL*.

## Supplementary Material

Crystal structure: contains datablocks global, I. DOI: 10.1107/S1600536810051445/xu5110sup1.cif

Structure factors: contains datablocks I. DOI: 10.1107/S1600536810051445/xu5110Isup2.hkl

Additional supplementary materials:  crystallographic information; 3D view; checkCIF report

## Figures and Tables

**Table 1:** Hydrogen-bond geometry (Å, °)

*D*—H⋯*A*	*D*—H	H⋯*A*	*D*⋯*A*	*D*—H⋯*A*
O1—H1⋯O2^i^	0.82	1.87	2.681 (7)	170

Symmetry code: (i) 
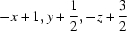
.

**Fig. 1.**
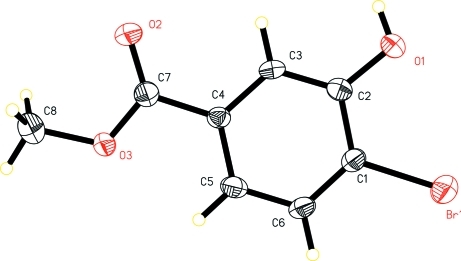
Title molecule showing the 30% probability displacement ellipsoids and the atom-numbering scheme.

**Fig. 2.**
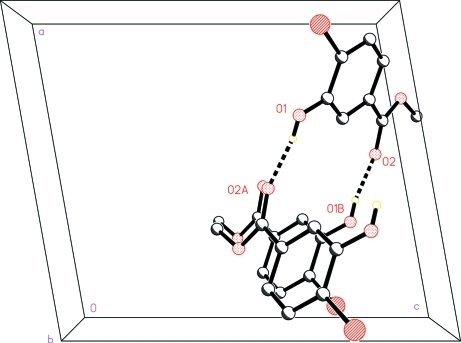
Crystal packing of the title compound as viewed along the b axis. Dashed lines indicate helical hydrogen-bonding chain Symmetry: A = -x + 1, y + 1/2, -z+1.5; B = -x + 1, y - 1/2, -z+1.5.

## supplementary crystallographic information

### Comment

The methyl 3-hydroxybenzoate derivatives as important starting materials have been applied to synthesis various broad-spectrum antimicrobials (Nie *et al.*, 2005) and design cryptophane derivatives in self-assembling of supermolecular chemistry (Zhong *et al.*, 2001). Here we report the structure of the title compound (Fig. 1).

In the crystal structure of the title compound, there are linked by an intermolecular O-H···O hydrogen bond between hydroxyl and carbonyl groups forming an infinite helical chain along the *b* axis (Table 1 and Fig. 2). And no significant interaction is observed between the chains.

### Experimental

The title compound was synthesized as previously reported (Nie *et al.*, 2005). The electrospray ionization mass spectrum (ESI-MS) showed an intense peak of molecular ions at *m*/*z* 230. Single crystals suitable for X-ray diffraction were obtained from methanol solution in room temperature.

### Refinement

H atoms were placed in calculated positions with O—H = 0.82 and C—H = 0.93-0.96 Å, and refined in riding mode with U_iso_(H) = 1.5U_eq_(O,C) for hydroxy H and methyl H atoms and 1.2U_eq_(C) for the others.

### Crystal data

**Table:**

C_8_H_7_BrO_3_	*F*(000) = 456
*M**_r_* = 231.05	*D*_x_ = 1.828 Mg m^−^^3^
Monoclinic, *P*2_1_/*c*	Mo *K*α radiation, λ = 0.71073 Å
Hall symbol: -P 2ybc	Cell parameters from 1334 reflections
*a* = 10.812 (4) Å	θ = 2.3–24.2°
*b* = 6.317 (2) Å	µ = 4.86 mm^−^^1^
*c* = 12.490 (5) Å	*T* = 293 K
β = 100.164 (6)°	Plate, colorless
*V* = 839.7 (5) Å^3^	0.27 × 0.24 × 0.16 mm
*Z* = 4	

### Data collection

**Table:**

Bruker SMART CCD 1000 diffractometer	1831 independent reflections
Radiation source: fine-focus sealed tube	1129 reflections with *I* > 2σ(*I*)
graphite	*R*_int_ = 0.042
Detector resolution: 10.0 pixels mm^-1^	θ_max_ = 27.2°, θ_min_ = 1.9°
φ and ω scans	*h* = −13→13
Absorption correction: multi-scan (*SADABS*; Sheldrick, 1996)	*k* = −8→3
*T*_min_ = 0.354, *T*_max_ = 0.511	*l* = −15→16
4755 measured reflections	

### Refinement

**Table:**

Refinement on *F*^2^	Primary atom site location: structure-invariant direct methods
Least-squares matrix: full	Secondary atom site location: difference Fourier map
*R*[*F*^2^ > 2σ(*F*^2^)] = 0.058	Hydrogen site location: difference Fourier map
*wR*(*F*^2^) = 0.172	H-atom parameters constrained
*S* = 1.08	*w* = 1/[σ^2^(*F*_o_^2^) + (0.0685*P*)^2^ + 2.8188*P*] where *P* = (*F*_o_^2^ + 2*F*_c_^2^)/3
1831 reflections	(Δ/σ)_max_ < 0.001
111 parameters	Δρ_max_ = 0.46 e Å^−^^3^
0 restraints	Δρ_min_ = −0.55 e Å^−^^3^

### Special details

**Table:**

Geometry. All esds (except the esd in the dihedral angle between two l.s. planes) are estimated using the full covariance matrix. The cell esds are taken into account individually in the estimation of esds in distances, angles and torsion angles; correlations between esds in cell parameters are only used when they are defined by crystal symmetry. An approximate (isotropic) treatment of cell esds is used for estimating esds involving l.s. planes.
Refinement. Refinement of F^2^ against ALL reflections. The weighted R-factor wR and goodness of fit S are based on F^2^, conventional R-factors R are based on F, with F set to zero for negative F^2^. The threshold expression of F^2^ > 2sigma(F^2^) is used only for calculating R-factors(gt) etc. and is not relevant to the choice of reflections for refinement. R-factors based on F^2^ are statistically about twice as large as those based on F, and R- factors based on ALL data will be even larger.

### Fractional atomic coordinates and isotropic or equivalent isotropic displacement parameters (Å^2^)

**Table:**

	*x*	*y*	*z*	*U*_iso_*/*U*_eq_	
Br1	0.92070 (8)	1.12616 (14)	0.78430 (7)	0.0617 (4)	
C1	0.8397 (6)	0.8935 (11)	0.8349 (5)	0.0392 (15)	
C2	0.7180 (6)	0.8391 (11)	0.7823 (5)	0.0382 (15)	
C3	0.6621 (5)	0.6667 (10)	0.8204 (5)	0.0357 (15)	
H3	0.5809	0.6296	0.7877	0.043*	
C4	0.7238 (6)	0.5454 (10)	0.9072 (5)	0.0331 (14)	
C5	0.8426 (6)	0.6045 (11)	0.9592 (5)	0.0425 (16)	
H5	0.8837	0.5274	1.0183	0.051*	
C6	0.8996 (6)	0.7797 (12)	0.9223 (6)	0.0449 (17)	
H6	0.9792	0.8206	0.9571	0.054*	
C7	0.6617 (6)	0.3570 (10)	0.9440 (5)	0.0396 (15)	
C8	0.6834 (8)	0.0661 (13)	1.0640 (6)	0.057 (2)	
H8A	0.6194	0.1164	1.1020	0.086*	
H8B	0.7478	−0.0043	1.1142	0.086*	
H8C	0.6471	−0.0314	1.0082	0.086*	
O1	0.6635 (5)	0.9620 (9)	0.6988 (4)	0.0565 (14)	
H1	0.5947	0.9128	0.6724	0.085*	
O2	0.5536 (5)	0.3093 (9)	0.9112 (4)	0.0589 (15)	
O3	0.7379 (4)	0.2443 (7)	1.0147 (4)	0.0432 (11)	

### Atomic displacement parameters (Å^2^)

**Table:**

	*U*^11^	*U*^22^	*U*^33^	*U*^12^	*U*^13^	*U*^23^
Br1	0.0603 (5)	0.0604 (6)	0.0619 (5)	−0.0167 (4)	0.0043 (4)	0.0095 (4)
C1	0.037 (3)	0.039 (4)	0.041 (3)	−0.006 (3)	0.006 (3)	−0.002 (3)
C2	0.037 (3)	0.043 (4)	0.033 (3)	0.003 (3)	0.003 (3)	−0.001 (3)
C3	0.026 (3)	0.043 (4)	0.037 (3)	−0.004 (3)	−0.001 (2)	−0.006 (3)
C4	0.033 (3)	0.035 (3)	0.030 (3)	−0.002 (3)	0.001 (3)	−0.003 (3)
C5	0.041 (4)	0.038 (4)	0.044 (4)	0.004 (3)	−0.008 (3)	−0.004 (3)
C6	0.037 (4)	0.043 (4)	0.050 (4)	−0.009 (3)	−0.003 (3)	−0.005 (3)
C7	0.045 (4)	0.036 (4)	0.037 (3)	−0.002 (3)	0.006 (3)	−0.005 (3)
C8	0.072 (5)	0.051 (5)	0.049 (4)	0.003 (4)	0.009 (4)	0.006 (4)
O1	0.050 (3)	0.057 (3)	0.055 (3)	−0.008 (3)	−0.011 (2)	0.022 (3)
O2	0.040 (3)	0.057 (3)	0.072 (3)	−0.014 (2)	−0.011 (2)	0.014 (3)
O3	0.044 (3)	0.037 (3)	0.044 (3)	−0.005 (2)	−0.003 (2)	0.009 (2)

### Geometric parameters (Å, °)

**Table:**

Br1—C1	1.876 (7)	C5—H5	0.9300
C1—C6	1.370 (10)	C6—H6	0.9300
C1—C2	1.406 (9)	C7—O2	1.206 (8)
C2—O1	1.349 (8)	C7—O3	1.307 (8)
C2—C3	1.371 (9)	C8—O3	1.457 (9)
C3—C4	1.397 (9)	C8—H8A	0.9600
C3—H3	0.9300	C8—H8B	0.9600
C4—C5	1.384 (9)	C8—H8C	0.9600
C4—C7	1.480 (9)	O1—H1	0.8200
C5—C6	1.385 (10)		
			
C6—C1—C2	121.0 (6)	C1—C6—C5	120.6 (6)
C6—C1—Br1	119.8 (5)	C1—C6—H6	119.7
C2—C1—Br1	119.2 (5)	C5—C6—H6	119.7
O1—C2—C3	124.5 (6)	O2—C7—O3	123.5 (6)
O1—C2—C1	117.7 (6)	O2—C7—C4	124.1 (6)
C3—C2—C1	117.7 (6)	O3—C7—C4	112.4 (5)
C2—C3—C4	121.7 (6)	O3—C8—H8A	109.5
C2—C3—H3	119.1	O3—C8—H8B	109.5
C4—C3—H3	119.1	H8A—C8—H8B	109.5
C5—C4—C3	119.5 (6)	O3—C8—H8C	109.5
C5—C4—C7	120.4 (6)	H8A—C8—H8C	109.5
C3—C4—C7	120.1 (5)	H8B—C8—H8C	109.5
C4—C5—C6	119.3 (6)	C2—O1—H1	109.5
C4—C5—H5	120.3	C7—O3—C8	116.9 (6)
C6—C5—H5	120.3		
			
C6—C1—C2—O1	178.1 (6)	C2—C1—C6—C5	1.7 (11)
Br1—C1—C2—O1	−1.8 (8)	Br1—C1—C6—C5	−178.5 (5)
C6—C1—C2—C3	−0.9 (10)	C4—C5—C6—C1	−0.3 (10)
Br1—C1—C2—C3	179.2 (5)	C5—C4—C7—O2	171.3 (7)
O1—C2—C3—C4	179.8 (6)	C3—C4—C7—O2	−7.8 (10)
C1—C2—C3—C4	−1.2 (9)	C5—C4—C7—O3	−10.2 (9)
C2—C3—C4—C5	2.6 (10)	C3—C4—C7—O3	170.7 (6)
C2—C3—C4—C7	−178.3 (6)	O2—C7—O3—C8	−7.1 (10)
C3—C4—C5—C6	−1.8 (10)	C4—C7—O3—C8	174.4 (5)
C7—C4—C5—C6	179.1 (6)		

### Hydrogen-bond geometry (Å, °)

**Table:**

*D*—H···*A*	*D*—H	H···*A*	*D*···*A*	*D*—H···*A*
O1—H1···O2^i^	0.82	1.87	2.681 (7)	170

Symmetry codes: (i) −*x*+1, *y*+1/2, −*z*+3/2.
